# Use of next-generation DNA sequencing to analyze genetic variants in rheumatic disease

**DOI:** 10.1186/s13075-014-0490-4

**Published:** 2014-11-22

**Authors:** Graham B Wiley, Jennifer A Kelly, Patrick M Gaffney

**Affiliations:** Arthritis and Clinical Immunology Program, Oklahoma Medical Research Foundation, 825 NE 13th Street, Oklahoma City, OK USA

## Abstract

Next-generation DNA sequencing has revolutionized the field of genetics and genomics, providing researchers with the tools to efficiently identify novel rare and low frequency risk variants, which was not practical with previously available methodologies. These methods allow for the sequence capture of a specific locus or small genetic region all the way up to the entire six billion base pairs of the diploid human genome.

Rheumatic diseases are a huge burden on the US population, affecting more than 46 million Americans. Those afflicted suffer from one or more of the more than 100 diseases characterized by inflammation and loss of function, mainly of the joints, tendons, ligaments, bones, and muscles. While genetics studies of many of these diseases (for example, systemic lupus erythematosus, rheumatoid arthritis, and inflammatory bowel disease) have had major successes in defining their genetic architecture, causal alleles and rare variants have still been elusive. This review describes the current high-throughput DNA sequencing methodologies commercially available and their application to rheumatic diseases in both case–control as well as family-based studies.

## Introduction

Within the past 6 years, the advent of high-throughput sequencing methodologies has provided researchers and clinicians with an extremely powerful tool for querying large amounts of the genetic landscape within not only single individuals but also cohorts of many individuals. Often dubbed ‘next-generation sequencing’ (NGS) or ‘second generation sequencing’ , these methodologies rely on the parallel processing of hundreds of thousands (if not hundreds of millions) of physically sequestered, individually (clonally) amplified copies of DNA, allowing for the generation of massive amounts of data in an extremely short period of time. The resulting datasets, which have become rich gold mines for researchers, provide catalogs of single nucleotide polymorphisms (SNPs), deletion/insertion polymorphisms, copy number variants, and translocations.

NGS DNA methodologies allow researchers to capture particular regions of interest contained within a genome or sequence the entire genome as a whole (whole-genome sequencing). Enriched regions may be specific loci or small genomic regions (targeted sequencing) or the sequences of all known genes and functional elements (exome sequencing). With each method having its own pros and cons, one must consider the scientific objective along with both cost and efficiency when choosing a method. One should not require, for example, the entirety of an exome to be sequenced if the functional variant in question is suspected to be in a non-coding region or previously implicated haplotype block. Similarly, the entire genome need not be sequenced if the study design is focusing only on variants affecting protein-coding genes. Finally, the amount of sequence generated per sample must be taken into account. NGS sequencers are currently optimized to output a set number of reads per run, generally far in excess of a single sample’s needs for adequate coverage. To effectively utilize this resource and decrease costs, researchers combine or ‘multiplex’ samples into shared lanes to reduce cost. This can, however, lead to a decrease in the overall number of reads per sample if the allocation is not meted out judiciously and result in reduced reliability of the calls due to insufficient coverage. Conversely, an overabundance of reads per sample may saturate coverage, diminishing returns on variant calling. Numbers of reads for a given sequence methodology have been empirically ascertained, beyond which increased sequence data yield little or no further variant information [1]. This may increase costs unnecessarily, resulting in fewer samples run for a given budget.

The major NGS platforms currently available to researchers and clinicians include Illumina’s HiSeq and MiSeq, Life Technologies’ Ion Torrent and SOLiD, and Roche’s 454. While the technologies empowering each of these platforms are quite different, with each having its own nuances in performance and powers of detection, they all rely on the ability to shear DNA into short (<1 kb) fragments, ligate adapters of known sequence to each end, and then immobilize and clonally amplify these molecules onto a solid substrate prior to undergoing massively parallel sequencing. An in-depth discussion of the pros and cons of each technology is beyond the scope of this review, but they are reviewed in other publications [2-4].

Today, these methodologies have revolutionized disease-gene discovery and are now being applied to genetics studies of rheumatic disease. While candidate gene and genome-wide association studies (GWASs) have had great success in identifying candidate genes for many of the rheumatic diseases (for example, >40 known genes in systemic lupus erythematosus (SLE) [5], >100 in rheumatoid arthritis (RA) [6], and >150 in inflammatory bowel disease (IBD) [7]), the extent of heritability explained by the majority of these genes remains small. DNA sequencing methodologies will surely result in additional gene identifications (especially rare variants that are not captured by GWAS methods) that may help explain missing heritability as well as shed light on structural variation within the genome.

## High-throughput genomic sequencing methodologies

**Figure 1:**
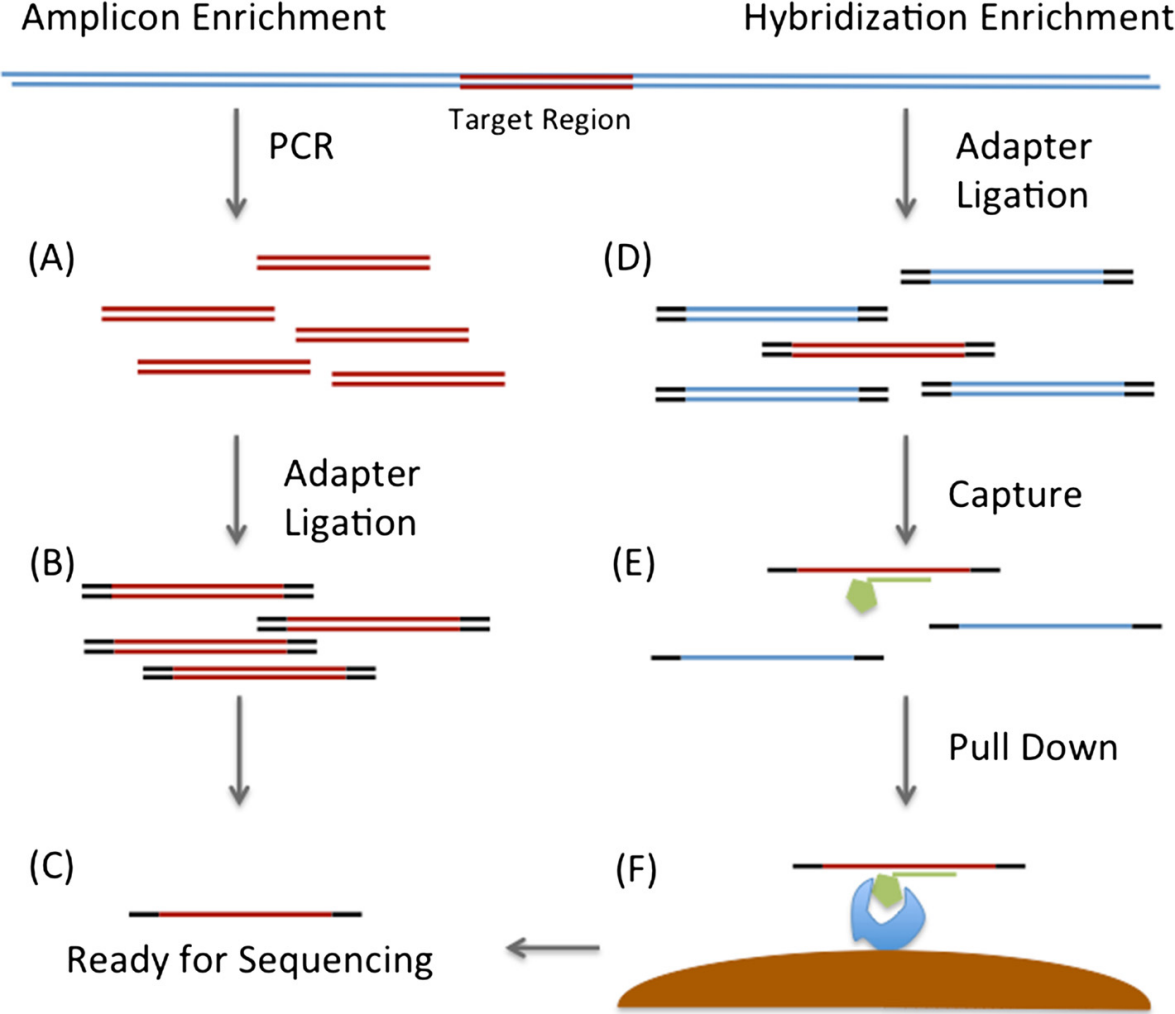
**A comparison of two popular sequence enrichment methods. (A)** For amplicon enrichment, PCR primers specific to the region of interest are used to amplify the target area. **(B)** These PCR products are then prepared for sequencing via ligation with sequencer-specific DNA molecules (adapters). **(C)** Molecules are then ready for sequencing. **(D)** For hybridization enrichment, the entire genome is sheared into small fragments which are subsequently ligated to sequencer-specific adapter DNA molecules. **(E)** Biotinylated oligomers that have been designed to be complementary to the region of interest are incubated with the previously generated sequencing library. **(F)** Captured molecules from the region of interest are pulled down using streptavidin-coated magnetic beads. DNA molecules are then eluted and ready for sequencing (C).

Targeted sequencing involves the enrichment of a certain locus or group of loci in a varying number of samples. The two most commonly used targeted sequencing approaches are based on either capture with complementary oligomers (hybridization) or amplification via PCR (amplicon) (Figure 1). Hybridization utilizes short biotinylated oligomers that have been designed, generally by an algorithm supplied by the reagent manufacturer, to tile over the locus/loci of interest. These ‘bait’ oligomers are hybridized to the genomic DNA sample and allow for the capture of their specific complementary DNA sequences. This approach is generally favored for large numbers of loci and has the ability to cover up to 20 million base pairs (Mbp) of target regions. Amplicon sequencing methods consist of primer-walking across the locus/loci of interest, followed by pooling the sometimes large number of PCR reactions prior to sequencing. This approach is primarily for regions up to 1 to 2 Mbp total, but allows for large numbers of samples to be pooled together in a single sequencing reaction. Targeted sequencing is often the method of choice for follow-up studies of GWAS associations. Its main disadvantage is that it is generally unable to perform well across repetitive elements within the genome, regions that have low-complexity, or extreme A-T or G-C sequence content.

Exome sequencing is, for all intents and purposes, the same as hybridization targeted capture in methodology. The differences lie in the fact that the exome capture systems have been specifically designed to only capture the coding regions of known genes and, in some cases, known functional non-coding elements of the genome. This optimization allows for a single exome capture system to enrich for 35 to 80 Mbp total. The goal in studying the exomes is to identify mutations that alter the amino acid content of a protein, possibly resulting in altered protein function. Exome capture systems may also include the untranslated regions of genes, pseudogenes, long non-coding RNAs, microRNA genes, and other genomic elements of interest that do not necessarily fall under the moniker of ‘gene’. The inclusion of these other loci is heavily dependent on the manufacturer and version of the exome capture system. Since it uses the same methods as targeted sequencing, exome capture technology also shares its disadvantages, with approximately 10% of the exome routinely failing to be captured and, thus, being unable to be sequenced.

Whole-genome sequencing allows for the potential identification of every variant in the genome. It is the most straightforward of the NGS methodologies since the entire genome is prepared and placed onto the sequencer with minimal processing. However, due to the large number of sequencing reads necessary to cover the entire genome, let alone the appropriate amount of coverage necessary to generate good quality variant calls, it remains the most expensive. For this reason very few rheumatic disease studies have yet undertaken whole-genome sequencing. However, we anticipate that this will not be the case for much longer since the cost for whole-genome sequencing continues to decrease.

While we provide below a few examples of how each DNA sequencing methodology has been applied to various rheumatic diseases, additional examples are included for the reader in Table 1.

**Table 1:** **Rheumatic disease studies utilizing next-generation DNA sequencing methodologies**

**Disease**	**Sequencing application**	**Platform**	**Study design**	**Sample size sequenced**	**Population**	**Associated genes**	**Reference**
AS	Targeted	Illumina	Case–control	50 cases and 50 controls; 846 cases and 1,308 controls	Han Chinese/ European	*IL23R*	[8]
BD	Exome	Illumina	Case–control	766 cases, 768 controls	Japanese; Turkish	*IL23R*, *TLR4*, *MEFV*	[9]
	Exome	Illumina	Case–control	32 cases, 59 controls	Korean	*KIR3DL3*, *MTHFR*, *MICA*, *KIR2KL4*, *FCGR3A*, *ICAM1*, *IFNAR1*, *KIR2DL4*	[10]
IBD	Exome	Illumina	Case–control	350 cases, 350 controls	Caucasian	*NOD2*, *IL23R*, *CARD9*, *IL18RAP*, *CUL2*, *C1orf106*, *PTPN22*, *MUC19*	[11]
	Sanger	Applied Biosystems	Case–control	528 cases, 549 controls	Caucasian	*TNFRSF6B*	[12]
CU	Whole genome/Sanger	Illumina/Applied Biosystems	Family	1 case; 27 cases	Caucasian	*PLCG2*	[13]
DDH	Whole exome	Illumina	Family	4 cases	Caucasian	*CX3CR1*	[14]
FMS	Whole exome	Illumina	Family	19 cases, 150 trios	Caucasian	*ZNF77*, *KNG1*, *MMP8*, *STARD6*, *C14orf105*, *FAM81B*, *C11orf40*	[15]
Gout	Whole genome	Illumina	Case–control	457 cases	Caucasian	*ALDH16A1*	[16]
HUVS	Whole exome/targeted exome	Illumina	Family	1 case; 3 cases, 9 unaffecteds	Caucasian	*DNASE1L3*	[17]
OA	Sanger	Applied Biosystems	Case–control	992 cases, 944 controls	Caucasian	*GDF5*	[18,19]
	Whole genome	Illumina	Case–control	623 cases, 69,153 controls	Icelandic	*ALDH1A2*	[20]
RA	Targeted	Beckman Coulter	Case–control	34 cases, 74 controls	Alaska Native	*MHC*	[21]
	Exome	Illumina	Case–control	500 cases, 650 controls	Caucasian	*CD2*, *IL2RA*, *IL2RB*	[22]
	Whole exome	Illumina	Case–control	19 cases	Japanese	*BTNL2*	[23]
	Whole exome/ targeted exome	Illumina	Consanguineous family/case–control	4 cases; 1,088 cases, 1,088 controls	Caucasian	*PLB1*	[24]
SLE	Targeted	Illumina	Case–control	74 cases, 100 controls	Caucasian	*UBE2L3*	[25]
	Targeted	Illumina	Case–control	100 cases, 100 controls	Caucasian	*IKBKE*, *IFIH1*	[26]
	Targeted	Illumina	Case–control	218 cases, 157 controls	Caucasian; African-American; Hispanic	*TNIP1*	[27]
	Targeted	Illumina	Case–control	7 cases	Caucasian	*TNFAIP3*	[28]
	Exome	Sanger	Case–control	191 cases, 96 controls	Caucasian	*FAM167A; BLK*	[29]
	Whole exome/Sanger	Life Technologies	Family	1 case; 3 cases, 3 unaffecteds	Caucasian	*PRKCD*	[30]
pSS	Targeted	Sanger	Case–control	19 cases	Caucasian	*TNFAIP3*	[31]

AS, ankylosing spondylitis; BD, Behçet’s disease; IBD, inflammatory bowel disease; CU, cold-induced urticaria; DDH, developmental dysplasia of the hip; FMS, fibromyalgia syndrome; HUVS, hypocomplementemic urticarial vasculitis syndrome; OA, osteoarthritis; RA, rheumatoid arthritis; SLE, systemic lupus erythematosus; pSS, primary Sjögren’s syndrome.

## Other sequencing methodologies

While not a main focus of this review, there are other high-throughput sequencing methods available to researchers that focus on non-genetic variation (epigenetics and transcriptomics). The epigenome consists of alterations resulting from environmental exposures to chemical, nutritional and physical factors that ultimately result in changes to gene expression, suppression, development, or tissue differentiation without altering the underlying DNA sequence. Epigenetic modifications can occur on DNA (methylation) or the histone proteins that compact DNA into nucleosomes (histone modification). Several rheumatic disease studies are already utilizing powerful methods to determine epigenetic influences on phenotype and are discussed in multiple reviews [32-35].

Deep sequencing for transcriptomic studies (RNA-seq) generates more detailed data, including specific isoform, exon-specific transcript and allelic expression levels [36-38], mapping of transcription start sites, identification of sense and antisense transcripts, detection of alternative splicing events, and discovery of unannotated exons [39,40]. To date, RNA-seq methods have been conducted in rheumatic disease studies of RA [41] and SLE [42,43], and in a murine model of inflammatory arthritis [44].

## Targeted DNA sequencing approach in rheumatic disease

A number of targeted deep sequencing studies for rheumatic diseases have been used to follow up associations identified by GWASs or custom designed genotyping arrays (Table 1) [25-28]. Adrianto and colleagues [27,28] have performed two such studies in SLE-associated risk loci, *TNFAIP3* and *TNIP1. TNFAIP3* was first identified as an SLE risk gene by GWAS and encodes the ubiquitin-modifying enzyme A20, which is a key regulator of NF-kB activity [45,46]. After confirming genetic association in a large case–control association study of five racially diverse populations, Adrianto and colleagues utilized a targeted sequencing approach of the associated *TNFAIP3* risk haplotype in seven carriers (two homozygotes and five heterozygotes) [28]. Though they did not identify any novel SNPs, they did identify a previously unreported single base deletion present on all risk chromosomes. This deletion was adjacent to a rare SNP found in Europeans and Asians and, together, this SNP-indel variant pair formed a TT > A polymorphic dinucleotide that bound to NF-kB subunits with reduced avidity. In addition, the risk haplotype that carried the TT > A variant reduced *TNFAIP3* mRNA and A20 protein expression. *TNIP1* (TNFAIP3 interacting protein 1) has also been associated with SLE in multiple studies, and in conjunction with their studies of *TNFAIP3*, Adrianto and colleagues [27] performed a similar targeted sequencing study of *TNIP1*. Targeted resequencing data resulted in 30 novel variants that were then imputed back into a large, ethnically diverse case–control study, and conditional analysis was used to identify two independent risk haplotypes within *TNIP1* that decrease expression of *TNIP1* mRNA and ABIN1 protein. In a similar fashion, S Wang and colleagues [25] conducted a targeted sequencing study of the SLE-associated *UBE2L3* locus in 74 SLE cases and 100 European controls. They identified five novel variants (three SNPs and two indels) that were not present in NCBI dbSNP build 132, one of which was strongly associated with SLE (*P* = 2.56 × 10^−6^). The variants were then imputed back into a large case–control dataset, which ultimately led to the identification of a 67 kb *UBE2L3* risk haplotype in four racial populations that modulates both *UBE2L3* and *UBCH7* expression.

C Wang and colleagues [26] explored the variants within and around *IKBKE* and *IFIH1*, genes also previously identified as associated with SLE. These two genes were targeted using an amplicon long-range PCR-based strategy of exonic, intronic, and untranslated regions in 100 Swedish SLE cases and 100 Swedish controls. In the course of their sequencing, they identified 91 high-quality SNPs in *IFIH1* and 138 SNPs in *IKBKE*, with 30% of the SNPs identified being novel. Putative functional alleles were then genotyped in a large Swedish cohort, which ultimately yielded two independent association signals within both *IKBKE* (one of which impairs the binding motif of SF1, thus influencing its transcriptional regulatory function) and *IFIH1*.

Davidson and colleagues [8] utilized targeted sequencing of the *IL23R* gene to identify rare polymorphisms associated with ankylosing spondylitis in a Han Chinese population. Targeted sequencing of a 170 kb region containing *IL23R* and its flanking regions was performed in 100 Han Chinese subjects and again in 1,950 subjects of European descent and identified several potentially functional rare variants, including a non-synonymous risk variant (G149R) that proved to be associated with the disease.

## Exome studies in rheumatic disease

Many studies have resequenced the exomes of candidate genes to identify variants that are likely to influence protein function and, thus, have biological relevance (Table 1) [9-11,22,29]. For example, Rivas and colleagues [11] utilized targeted exome resequencing to query 56 loci previously associated with IBD. They used an amplicon pooling strategy in 350 IBD cases and 350 controls and identified 429 high confidence variants, 55% of which were not included in dbSNP. Seventy rare and low-frequency protein-altering variants were then genotyped in nine independent case–control datasets comprising 16,054 Crohn’s cases, 12,153 ulcerative colitis cases, and 17,575 controls, which identified previously unknown associated IBD risk variants in *NOD2*, *IL18RAP*, *CUL2*, *C1orf106*, *PTPN22*, and *MUC19*. They also identified protective variants within *IL23R* and *CARD9*. Their results were among the first to support the growing hypothesis that common, low-penetrance alleles as well as rare, highly penetrant alleles can exist within the same gene. Other studies have taken a whole exome sequencing approach to target and evaluate all known exonic regions throughout the genome [23].

A primary benefit of these DNA methodologies is the ability to capture rare and low frequency variants that, until now, were unknown. With low frequency variants, however, the power of the widely used indirect linkage disequilibrium-mapping approach is low. Therefore, several studies have performed large-scale targeted exome sequencing studies using genetic burden testing, a method that evaluates the combined effect of an accumulation of rare and low-frequency variants within a particular genomic segment such as a gene or exon. Diogo and colleagues [22] applied this strategy to the exons of 25 RA genes discovered by GWAS while utilizing four burden methods and identified a total of 281 variants (83% with minor allele frequency <1% and 65% previously undescribed), with an accumulation of rare nonsynonymous variants located within the *IL2RA* and *IL2RB* genes that segregated only in the RA cases. Eleven RA case–control dense genotyping array datasets (ImmunoChip and GWAS) comprising 10,609 cases and 35,605 controls were then scrutinized for common SNPs that were in linkage disequilibrium with the 281 variants identified by the exome sequencing. Sixteen of 47 identified variants were subsequently associated with RA, demonstrating that, in addition to previously known common variants, rare and low-frequency variants within the protein-coding sequence of genes discovered by GWASs have small to moderate effect sizes and participate in the genetic contribution to RA. Kirino and colleagues [9] also utilized burden testing while studying the exons of 10 genes identified through GWAS that were associated with Behçet’s disease and 11 known innate immunity genes in Japanese and Turkish populations. They used three different burden tests and were able to identify a statistically significant burden of rare, non-synonymous protective variants in *IL23R* (G149R and R381Q) and *TLR4* (D299G and T399I) in both populations, and association of a single risk variant in *MEFV* (M694V) within the Turkish population.

## Whole-genome sequencing in rheumatic disease

Until only recently, whole-genome sequencing was an unrealistic option for most studies due to its high costs. Today, however, with a cost approaching $1,000 per sample [47], genetics and genomics researchers are finally able to see this method as a valid option for their studies. To date, few published large-scale whole-genome sequencing studies have been conducted on a rheumatic disease. Sulem and colleagues [16] carried out the first such study, sequencing 457 Icelanders with various neoplastic, cardiovascular and psychiatric conditions to an average depth of at least 10× and identified approximately 16 million variants. These variants were then imputed into a chip-genotyped dataset of 958 gout cases and >40,000 controls with more than 15,000 of these subjects also having measured serum uric acid levels. When analyzing gout as the phenotype, two loci reached genome-wide significance: a novel association with an exonic SNP in *ALDH16A1* (*P* = 1.4 × 10^−16^), and a Q141K variant within *ABCG2* (*P* = 2.82 × 10^−12^), a gene previously reported to be associated with gout and serum uric acid levels. The *ALDH16A1* SNP displayed stronger association with gout in males and was correlated with a younger age at onset. Four loci reached genome-wide significant association when evaluating association with serum uric acid levels: the same *ALDH16A1* SNP found with gout (*P* = 4.5 × 10^−21^), a novel association with the chromosome 1 centromere (*P* = 4.5 × 10^−16^), as well as previously reported signals at *SLE2A9* (*P* = 1.0 × 10^−80^) and *ABCG2* (*P* = 2.3 × 10^−20^). Another study, by Styrkarsdottir and colleagues [20], utilized whole-genome sequencing of an Icelandic population to further inform a GWAS investigating severe osteoarthritis of the hand. In this case, the imputation of 34.2 million SNPs identified via whole-genome sequencing of 2,230 Icelandic subjects into a previously performed GWAS of 632 cases and 69,153 controls allowed the researchers to identify association with 55 common (41 to 52%) variants within a linkage disequilibrium block containing the gene *ALDH1A2* and four rare (0.02%) variants at 1p31. Other rheumatic disease studies have conducted much smaller scale whole-genome sequencing in one to five individuals followed by targeted exome or Sanger sequencing of the identified variants in larger samples [13].

## DNA sequencing in families with rheumatic disease

For rheumatic diseases showing an autosomal dominant or Mendelian inheritance pattern, the study of each genome across multiple generations of the same family can shed light on the variant(s) or gene(s) responsible for disease. Therefore, high-throughput DNA sequencing studies are not limited just to disease cases and population controls, but have been applied to family studies as well [13,14,17,24]. Okada and colleagues [24] recently applied whole-exome sequencing to a four-generation consanguineous Middle Eastern pedigree in which 8 of 49 individuals (16.3%) were affected with RA, which was much higher than the prevalence of RA in the general Middle Eastern population (1%). By applying a novel non-parametric linkage analysis method to GWAS data that looked for regional IBD stretches with a loss of homozygous genotypes in affected cases, they identified a 2.4 Mb region on 2p23 that was enriched in the RA cases. Whole-exome sequencing of 2p23 was performed in four RA cases, which identified a novel single missense mutation within the *PLB1* gene (c.2263G > C; G755R). Variants near the *PBL1* gene were then evaluated in 11 GWAS datasets of 8,875 seropositive RA cases and 29,367 controls, which identified two independent intronic mutations that, when evaluated as a haplotype, demonstrated significant association with RA risk (*P* = 3.2 × 10^−6^). Finally, deep exon sequencing of *PBL1* was performed in 1,088 European RA cases and 1,088 European controls, and burden testing revealed an enrichment of rare variants within the protein-coding region of *PBL1.* Taken together, these results suggest both coding and non-coding variants of *PBL1*, a gene that encodes both phopholipase A1 and A2 enzymatic activities, contribute to RA risk.

A major benefit of utilizing NGS methods within families is that researchers are now able to combine previously generated linkage information with new sequence data to identify rare causal variants that contribute to previously detected linkage signals.

Ombrello and colleagues [13] integrated NGS data with previously generated linkage data in three families with a dominantly inherited complex of cold-induced urticaria, antibody deficiency, and autoimmunity. Previous linkage analysis identified a 7.7 Mb interval on chromosome 16q21. Whole-genome sequencing of one affected individual from the first family did not identify any novel mutations within the linkage peak. When analyzing a second family, however, a segregated haplotype containing 24 genes overlapped a linkage interval, and *PLCG2* was subsequently chosen as the most likely candidate*.* Sequencing of *PLCG2* within family 1 identified a 5.9 kb deletion of exon 19 that was present only in the affected individuals. A *post hoc* analysis of the whole genome data from the family 1 individual confirmed the presence of this deletion. Subsequent sequencing of this gene in the other two families identified further deletions: transcripts in family 2 that lacked exons 20 to 22 because of an 8.2 kb deletion, and deletion of exon 19 in family 3 because of a 4.8 kb deletion. Each of the three deletions affected the carboxy-terminal Src-homology 2 (cSH2) domain of *PLCG2*, a domain that, in healthy individuals, couples the enzymatic activity of *PLCG2* to upstream pathways. In these individuals, however, the deletions resulted in auto-inhibition and constitutive phospholipase activity.

## Sanger sequencing in rheumatic disease

Until the application of NGS, Sanger sequencing, which was developed in 1977, was the most widely used sequencing method. However, the advent of NGS does not necessarily ring the death-knell for Sanger sequencing for one or a handful of variants. While on the wane as a large-scale experimental technique, this tried and true methodology still retains usefulness and economy in large-scale replication and screening assays. Many still consider this method to be the ‘gold standard’ and will utilize Sanger sequencing to validate the results generated by their high-throughput sequencing methods [20,23,24,30]. In addition, recently published studies have applied no other method but Sanger sequencing for deep sequencing of extremely specific regions in smaller numbers of samples. These include a search for rare variants across *GDF5*, a gene harboring a known susceptibility variant for osteoarthritis in 992 cases and 944 controls [18,19], a similar rare variant screen focused on *TNFRSF6B* in pediatric-onset IBD [12], exome sequencing of *TNFAIP3* in 19 primary Sjögren’s syndrome patients with lymphoma [31], and targeted sequencing of the *FAM167* and *BLK* exomes in 191 SLE cases and 96 controls [29].

## The future of sequencing

While a tried and true advancement in the genetics and genomics of rheumatic disease studies, deep sequencing, as a technological field, has and will continue to remain in a state of flux. With the continued refinement of technology and methods, sequencing costs have dropped tremendously over the past 5 years and, as of the drafting of this manuscript, whole-genome sequencing of humans has dropped to less than $1,000 per sample [48]. At this price point, the continued viability of exome sequencing as a widespread technique has yet to be determined. Indeed, it is quite within the realm of possibility that all patients will have their genomes sequenced as a routine test at presentation to their healthcare provider. The foreseeable rise of nanopore sequencers and other ‘third-generation’ sequencers able to process single molecules of DNA may make bedside sequencing a reality.

## Note

 This article is part of the series ‘*New technologies’*. Other articles in this series can be found at http://arthritis-research.com/series/technology.

## Abbreviations

GWAS: Genome-wide association study
IBD: Inflammatory bowel disease
Mbp: Million base pairs
NGS: Next-generation sequencing
PCR: Polymerase chain reaction
RA: Rheumatoid arthritis
SLE: Systemic lupus erythematosus
SNP: Single nucleotide polymorphism
